# Transcatheter Aortic Valve Replacement in Degenerated Perceval Bioprosthesis: Clinical and Technical Aspects in 32 Cases

**DOI:** 10.3390/jcm12196265

**Published:** 2023-09-28

**Authors:** Giovanni Concistrè, Tommaso Gasbarri, Marcello Ravani, Anees Al Jabri, Giuseppe Trianni, Giacomo Bianchi, Rafik Margaryan, Francesca Chiaramonti, Michele Murzi, Enkel Kallushi, Egidio Varone, Simone Simeoni, Alessandro Leone, Andrea Farneti, Sergio Berti, Marco Solinas

**Affiliations:** 1 Department of Adult Cardiac Surgery, G. Pasquinucci Heart Hospital, Fondazione CNR—G. Monasterio, 54100 Massa, Italy; gasbarri@ftgm.it (T.G.); gbianchi@ftgm.it (G.B.); margaryan@ftgm.it (R.M.); chiaramonti@ftgm.it (F.C.); michele.murzi@ftgm.it (M.M.); kallushi@ftgm.it (E.K.); varone@ftgm.it (E.V.); simeoni@ftgm.it (S.S.); aleone@ftgm.it (A.L.); farneti@ftgm.it (A.F.); solinas@ftgm.it (M.S.); 2 Department of Cardiology, G. Pasquinucci Heart Hospital, Fondazione CNR—G. Monasterio, 54100 Massa, Italy; ravanim@ftgm.it (M.R.); aljabri@ftgm.it (A.A.J.); trianni@ftgm.it (G.T.); ifcberti@ftgm.it (S.B.)

**Keywords:** aortic valve replacement, aortic bioprosthesis degeneration, sutureless aortic valve, transcatheter aortic valve

## Abstract

Background: Sutureless aortic bioprostheses are increasingly being used to provide shorter cross-clamp time and facilitate minimally invasive aortic valve replacement. As the use of sutureless valves has increased over the past decade, we begin to encounter their degeneration. We describe clinical outcomes and technical aspects in patients with degenerated sutureless Perceval (CorCym, Italy) aortic bioprosthesis treated with valve-in-valve transcatheter aortic valve replacement (VIV-TAVR). Methods: Between March 2011 and March 2023, 1310 patients underwent aortic valve replacement (AVR) with Perceval bioprosthesis implantation. Severe bioprosthesis degeneration treated with VIV-TAVR occurred in 32 patients with a mean of 6.4 ± 1.9 years (range: 2–10 years) after first implantation. Mean EuroSCORE II was 9.5 ± 6.4% (range: 1.9–35.1%). Results: Thirty of thirty-two (94%) VIV-TAVR were performed via transfemoral and two (6%) via transapical approach. Vascular complications occurred in two patients (6%), and mean hospital stay was 4.6 ± 2.4 days. At mean follow-up of 16.7 ± 15.2 months (range: 1–50 months), survival was 100%, and mean transvalvular pressure gradient was 18.7 ± 5.3 mmHg. Conclusion: VIV-TAVR is a useful option for degenerated Perceval and appears safe and effective. This procedure is associated with good clinical results and excellent hemodynamic performance in our largest single-center experience.

## 1. Introduction

Surgical aortic valve replacement (AVR) remains the gold standard treatment for patients with aortic valve disease [1,2]. The operative risk of AVR has improved in the last years, with a reduction of mortality from 4.3% to 2.6% [3,4]. Despite these results, elderly high-risk patients referred for AVR still have bad outcomes and, therefore, might benefit from sutureless technology in order to reduce morbidity and mortality [5,6,7,8,9,10]. In the last decade, sutureless aortic valve implantation has gained interest because of the rapid development of new prosthesis technologies. The Perceval aortic valve (CorCym, Saluggia, Italy) is a sutureless bioprosthesis, and several reports have shown mid and long-term follow-up results and some cases of valve degeneration [9,10,11,12,13,14,15].

Transcatheter aortic valve replacement (TAVR) is a good option for replacing degenerated surgical valves with a valve-in-valve (VIV) procedure. However, experience of VIV-TAVR for degenerated Perceval valves remains limited [11,12,13,14,15]. We present our experience of 32 degenerated Perceval in 1310 patients who underwent AVR with sutureless bioprosthesis implantation. The aim of this study was to report technical procedural aspects, clinical outcomes and mid-term follow-up of the largest single-center series of patients undergoing VIV-TAVR.

## 2. Materials and Methods

### 2.1. Patients

Between March 2011 and March 2023, 1310 patients with severe aortic stenosis underwent AVR at Ospedale del Cuore, Massa, Italy, with Perceval sutureless aortic bioprosthesis. Patient selection for this type of device was left to the discretion of the surgeon. Exclusion criteria were irregular aortic annulus or ascending aorta geometry and acute endocarditis. The ratio between the diameter of the superior annulus and the diameter of the sinotubular junction should not exceed 1.3. This ratio was determined with echocardiography and a CT scan. In all patients undergoing isolated AVR, we performed a CT scan to plan minimally invasive surgery. Informed consent was obtained from each patient, and the study protocol conforms to the ethical guidelines of the 1975 Declaration of Helsinki as reflected in a priori approval by the institution’s human research committee. Trivial and moderate paravalvular leakage is defined by Akins CW et al. and Généreux P et al. [16,17]. Transthoracic echocardiography was performed preoperatively, postoperatively, and at follow-up. Forty patients needed reintervention for structural Perceval bioprosthesis degeneration at mean years of 6.4 ± 1.9 years (range: 2–10 years). Eight patients underwent redo surgery with sutured bioprosthesis implantation and 32 patients underwent VIV-TAVR. Six of the eight operated patients were excluded from VIV-TAVR due to the absence of peripheral vascular access suitable for the interventional procedure and 2 due to the aortic annulus being too large to implant a balloon-expandable valve. In all patients, the first visit was made after 1 month, the second after 3 months, the third after one year and then one visit every year. Mean follow-up was 16.7 ± 15.2 months (range: 1–50 months) and it was 100% complete.

### 2.2. Technology

Perceval is a bovine pericardium prosthesis assembled on a nitinol stent. This bioprosthesis can be positioned by means of a dedicated delivery system. The delivery system guided the collapsed stent-mounted valve to the correct position by 3 guiding sutures (4/0 polypropylene) positioned at the nadir of each resected cusp. Once the delivery system is in position, the prosthesis is deployed, the guiding sutures are removed, and the valve is in place; post-dilation modeling is performed with a balloon (30 s at a pressure of 4 Atmosphere), and the valve is flushed with warm saline to optimize final sealing. The Perceval valve is available in sizes S, M, L, and XL. When there is no indication of oral anticoagulation therapy, we instituted 100 mg daily of aspirin.

### 2.3. VIV-TAVR Planning and Technical Procedures

**Figure 1:**
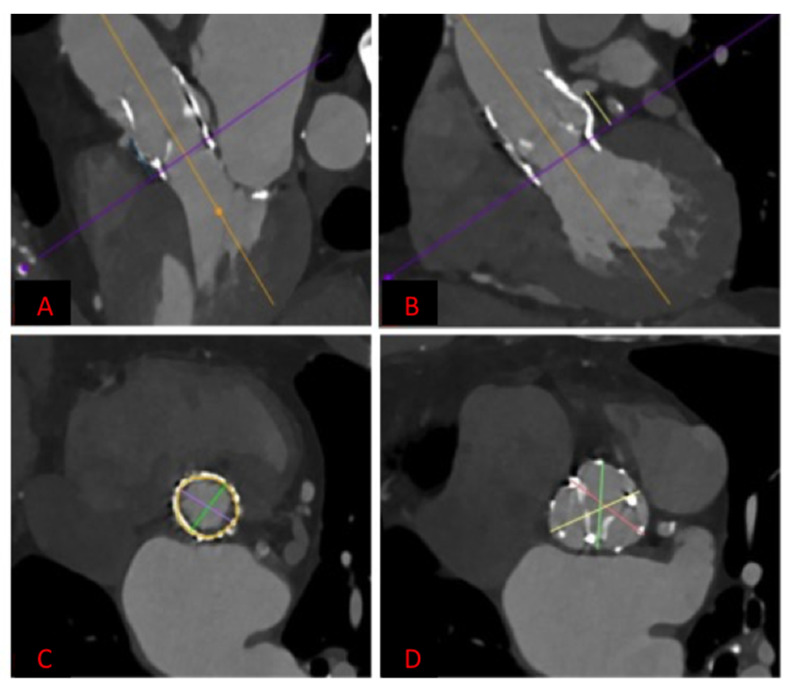
Preprocedural planning with ECG gated medium contrast CT scan. (**A**) Sizing of the landing zone (Perceval inflow ring) diameters, area and perimeter; (**B**) aortic root dimensions; (**C**) right coronary artery take off; (**D**) left coronary artery take off.

**Figure 2:**
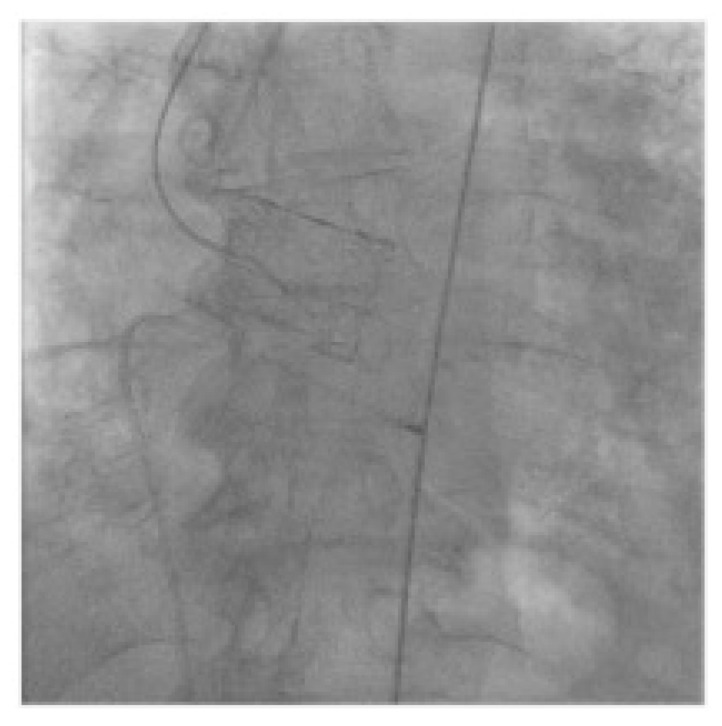
Crossing degenerated Perceval with AL catheter and straight tip guide wire.

**Figure 3:**
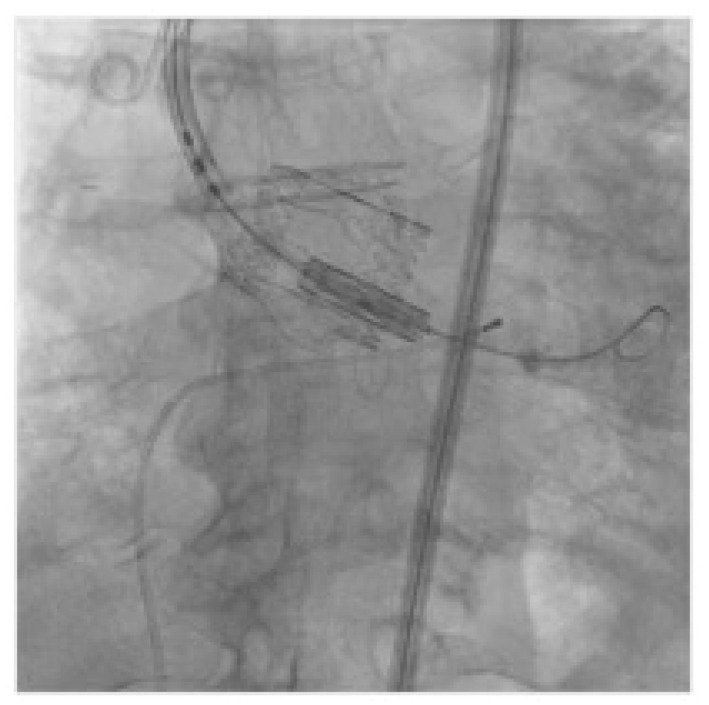
Crimped THV proper positioning.

**Figure 4:**
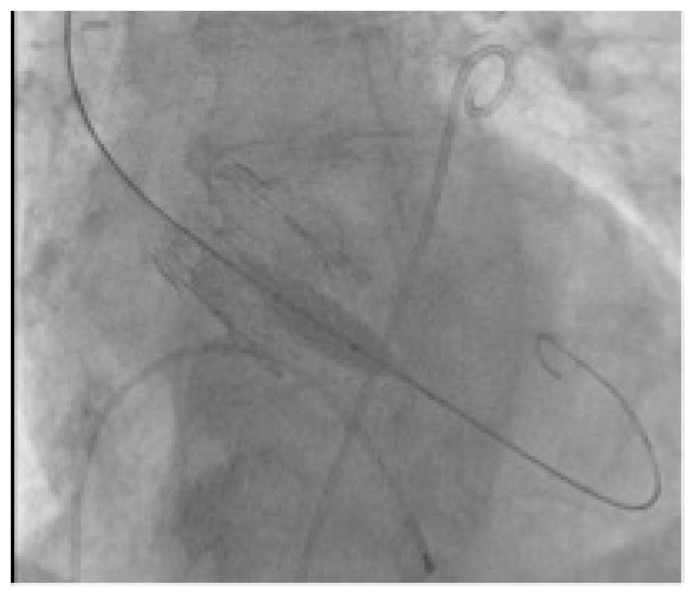
Soft predilatation and flossing.

**Figure 5:**
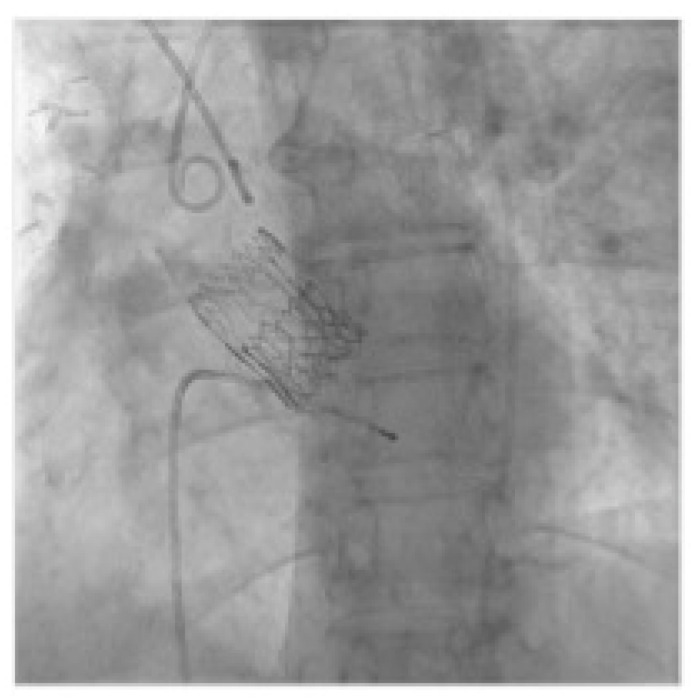
Final result.

Each patient with indication to replacement of degenerated Perceval underwent a preprocedural ECG-gated medium contrast CT scan to assess femoral artery dimensions and quality; delivery route quality (Figure 1A); degenerated bioprosthesis analysis; aortic root and coronary artery anatomical study (Figure 1B–D). In particular, the screening was focused on the presence of infolding, landing zone dimension (min and max diameters, perimeter and area), sinuses of Valsalva dimensions, and coronary artery height. The area measured at the level of the transcatheter heart valve (THV) landing zone, which corresponds to the Perceval inflow ring, is used to choose the correct expandable balloon THV size, according to the THV sizing chart. The procedure is conducted as a standard THV implantation and, in the case of femoral delivery, it is performed under local anesthesia during continuous hemodynamic monitoring and fluoroscopic guidance. A 6 French (F) pig-tail catheter is advanced in the ascending aorta through a right or left radial artery access for systemic arterial pressure monitoring and for performing aortography. Echo-guided puncture of the common femoral artery and femoral vein is obtained, and 6 F and 8 F sheath are, respectively, advanced. Through the venous access, a provisional 6 F pacer lead is placed in the right ventricle. Two Perclose^TM^ ProStyle^TM^ closure devices are pre implanted on the common femoral artery. Degenerated bioprosthesis is crossed by the use of an Amplatz Left catheter and a straight tip 0.038 guidewire (Figure 2). To help this step of the procedure, a hydrophilic 0.038 straight tip guide wire is used in case of a very high trans Perceval gradient, horizontal aorta, or any case of crossing difficulties. Careful attention is taken to avoid crossing the degenerated leaflet from the outside of the Perceval valve outflow ring. A 0.035 extra-stiff wire is then exchanged and placed in the left ventricle. Subsequently, a balloon expandable THV (Edwards Sapien 3) is implanted inside the degenerated bioprosthesis during rapid ventricular pacing. The Perceval valve inflow ring is used as a landing zone for THV implantation, aiming to align the proximal edge of the expanded THV to the proximal edge of the degenerated bioprosthesis (Figure 3). The THV size is chosen, matching the THV sizing chart and preprocedural CT scan measurements. As for native aortic valves, a 10% volume overfilling is admitted to adapt to the anatomy in case of borderline measures. In case of a very high trans Perceval gradient, horizontal aorta, or severe vascular tortuosity, a predilatation of the degenerated bioprosthesis and/or flossing with a small balloon is used to facilitate the crossing of the stenotic leaflet with the crimped THV. Flossing is also used to confirm proper crossing inside the Perceval outflow ring (Figure 4). Transthoracic echocardiography, fluoroscopy, angiography and invasive hemodynamic pressure measurement are used to asses THV function, hemodynamic performance and correct positioning (Figure 5) to check LV function and to exclude the presence of paravalvular leak and new onset of pericardial effusion. In the absence of post implant atrio-ventricular block, temporary pacer lead is removed. The THV sheath is removed, and hemostasis is obtained by the use of pre implanted closure devices. Vascular ultrasound and control aortography are used to show the patency of the ilio–femoral axis and the absence of bleeding. In the case of the transapical approach, the procedure is conducted under general anesthesia during continuous hemodynamic monitoring and fluoroscopic and transesophageal echo guidance. The left ventricular apex is identified, merging CT scan imaging and transthoracic echo imaging. Subsequently, a 6 to 7 cm antero-lateral thoracotomy is performed. A soft-tissue retractor and rib spreader are used to enhance the apex visibility. The pericardium is opened. The left ventricular apex puncture site is identified, avoiding the coronary artery by direct view and correct positioning is confirmed under echo check, palpating the apex. Two concentric 3/0 prolene pledgeted purse-string sutures are placed. The procedure is carried on as a standard transapical TAVR. In this case, due to the antegrade approach, the degenerated prosthesis is easily and quickly crossed with a standard 0.38 J tip guidewire. An extra stiff wire is exchanged, and the procedure is then continued with direct THV delivery as previously described (with proper THV orientation) without the need for predilatation and lossing. At the end of the procedure, the delivery system and the sheath are removed, and apex hemostasis is achieved by closing the two purse strings. The pericardium is closed over the ventricle, drainage is left in the pleural space and the chest is closed in a standard fashion.

The first reason for choosing Sapien transcatheter prosthesis is that in our Institution, we have developed a great experience in using the Sapien platform, and it was a natural consequence to treat a new degenerated prosthesis such as Perceval using a device with which we were very confident. Secondly, due to its radial force, the use of an expandable balloon valve is a perfect solution to treat an elliptical or distorted annulus, such as what happens in a degenerated Perceval; as we have shown, the Perceval itself can become circular and over-expands up to 2.5 mm, probably ending in a better hemodynamic performance. Eventually, we wanted to use a short frame valve in order to avoid any interference between the outflow ring of Perceval and the distal crown of any self-expandable valve, which can cause stent creep and lead to THV failure.

### 2.4. Statistical Analysis

Categorical variables are expressed as percentages; all continuous variables are expressed as mean ± standard deviation. The level of significance was set for *p* < 0.05 to reject the null hypothesis. We used the R statistical package (R core team; R Foundation for Statistical Computing, Vienna, Austria).

## 3. Results

In the selected period, thirty-two patients underwent VIV-TAVR in a degenerated Perceval Sutureless valve. All patients had preoperative “pure” aortic bioprosthesis stenosis (19%), steno-regurgitation (69%) and “pure” regurgitation (12%); the preoperative mean pressure trans-bioprosthesis gradient was 53.1 ± 18.3 mmHg. The mean EuroSCORE II was 9.5 ± 6.4%, and the mean STS score was 8.7 ± 4.2%.

A transfemoral approach was performed in thirty (94%) patients and a transapical approach in two (6%) patients due to the absence of suitable peripheral vascular accesses in one patient and due to concomitant VIV-transcatheter mitral valve replacement (TMVR) in the other patient. Baseline characteristics are shown in Table 1. The sizes of the degenerated Perceval were S (*n* = 5), M (*n* = 11), L (*n* = 14) and XL (*n* = 2). Concomitant procedures were performed in two patients (6%): one patient underwent concomitant percutaneous transluminal angioplasty of the left circumflex branch, and one patient concomitant VIV-TMVR (Table 2). The sizes of the Edwards Sapien transcatheter valve implanted were 20 mm (*n* = 6), 23 mm (*n* = 14), 26 mm (*n* = 11), and 29 mm (*n* = 1). Two patients had vascular complications: one patient had a common femoral artery pseudoaneurysm, and one patient had a common femoral artery dissection; both were treated with vascular stent implantation. The 30-day mortality was 0. The mean hospital stay was 4.6 ± 2.4 days. No patient required pacemaker implantation (Table 3). 

At a mean follow-up of 16.7 ± 15.2 months (range: 1–50 months), survival was 100%. One patient had a stroke, confirmed by computer tomography. Freedom from reintervention was 100%. Echocardiographic findings of 32 degenerated Perceval are shown in Table 4. Sapien prosthesis function and hemodynamic performance were assessed at discharge and at follow-up. The mean pressure gradient decreased significantly from a preoperative value of 53.1 ± 18.3 mmHg to 18.7 ± 5.3 mmHg at follow-up (*p* < 0.001). At follow-up, the left ventricular ejection fraction increased from 53.2 ± 8% to 56.2 ± 5.4% (*p* < 0.001). Moderate paravalvular leakage occurred in one patient with no clinical or laboratory signs of hemolysis and did not require any treatment; trivial paravalvular leakage was present in one patient at follow-up (Table 5).

## 4. Discussion

This study shows technical procedural aspects of clinical and echocardiographic results with VIV-TAVR in 32 degenerated Perceval sutureless aortic bioprosthesis. Our data show that the implantation of THV in Perceval is a safe and feasible procedure associated with a low complication rate and excellent hemodynamic performance at mid-term follow-up. Different from previous small reports [11,12,13,14,15], this study analyzed the largest single-center series of patients undergoing VIV-TAVR and mid-term follow-up.

**Figure 6:**
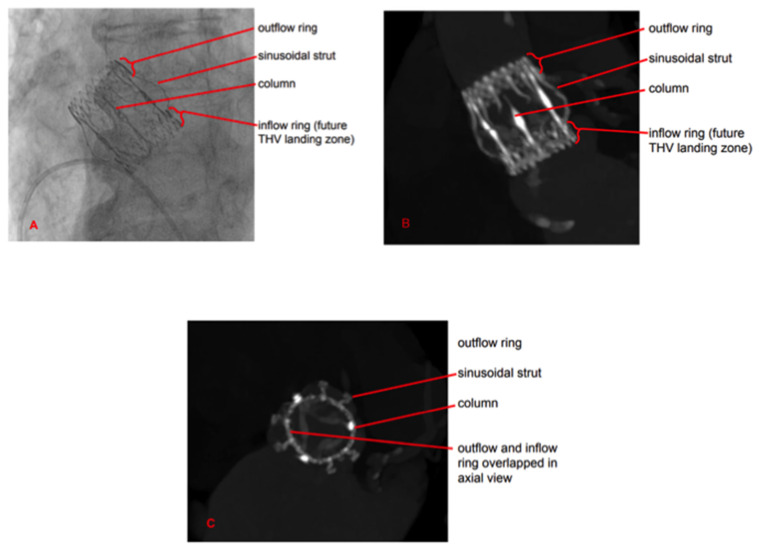
Radiological anatomy of Perceval. The Ni-Ti alloy radiopaque stent is clearly visible. (**A**) Fluoroscopic view; (**B**) CT scan long axis view; (**C**) CT scan short axis view.

**Figure 7:**
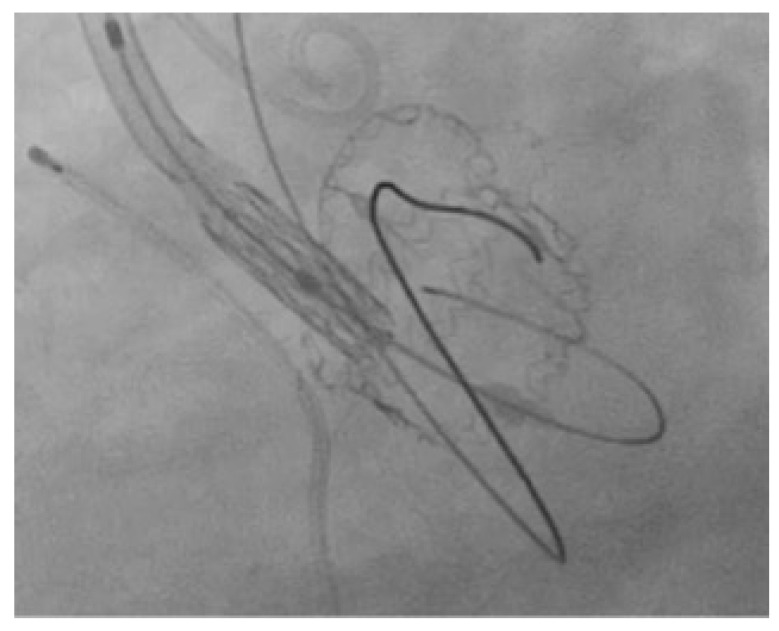
A case of wrong Perceval crossing outside the outflow ring, laterally and then inside the inflow ring with impossibility to advance and deliver the THV.

Sutureless bioprosthesis represents an innovative approach for surgical AVR and has been designed to allow faster implantation, reducing CPB and ACC time. This is an advantage for all patients, regardless of the risk profile. Therefore, Sutureless aortic valve implantation might be an alternative treatment option for patients at high-risk for mortality and morbidity after open heart surgery. The follow-up of this bioprosthesis is now long, and there are some cases of valve degeneration [9,10,11,12,13,14,15]. In our opinion, oversizing the prosthesis Perceval valve will not reduce the incidence presence of leakage; conversely, it might be associated with incomplete expansion dilatation of the bioprosthesis sutureless valve with infoldings of the annular portion. Sizing for the device is important as the Perceval is designed to expand to an outer diameter larger than the patient’s measured annular diameter. The expansion of the frame provides the proper interference fit to secure the Perceval in place for stability at physiological pressure, flow and movement. The Perceval bioprosthesis selected for the implant should match the measured diameter of the aortic annulus diameter. Margaryan et al. analyzed 54 patients who underwent Perceval implantation with preoperative contrast-enhanced multidetector-row computed tomography (MDCT). Echocardiographic measurements showed lower accuracy compared to MDCT measurements. MDCT measurements showed higher accuracy compared to echocardiographic measurements. They concluded that, possibly for precise aortic annulus measurement, contrast-enhanced MDCT is preferable [18]. They concluded that MDCT is possibly preferable for precise aortic annulus measurements. Sizing for the device is important as the Perceval is designed to expand to an outer diameter larger than the patient’s measured annular diameter. In a study of our Center, Cerillo et al. investigated the relationship between a computed tomography measure of the degree of prosthesis oversizing and the early hemodynamic and clinical outcomes in patients undergoing AVR with Perceval valve. The degree of oversizing of the implanted sutureless prosthesis was calculated as the ratio between the patients’ aortic annulus cross-sectional area and the ex vivo cross-sectional area of the implanted prosthesis in 151 Perceval patients who underwent preoperative cardiac computed tomography. This value was then entered in a multivariate analysis to ascertain its role as a predictor of early degeneration and increased the postoperative gradient and, therefore, early degeneration. The degree of oversizing of the implanted prosthesis was the most important predictor of increased postoperative prosthesis gradient (odds ratio, 1.264; 95% confidence interval, 1.147 to 1.394; odds ratio, 1.264; *p* < 0.0001). Interestingly, other relevant factors, such as patients’ body surface area, prosthesis size or patients’ body surface area, were not associated with increased transvalvular gradients. This study demonstrates that excessive oversizing in Perceval patients should be avoided and suggests that a different sizing planning algorithm, possibly based on cardiac computed tomography CT scan, should be developed [19]. Based on this study, since 2017, we have changed our policy, avoiding oversizing and basing the choice of bioprosthesis Perceval size above all with CT data, confirmed with intraoperative sizing. In our experience, 40 patients needed reintervention for structural Perceval bioprosthesis degeneration. Eight patients underwent redo surgery and 32 patients VIV-TAVR. The condition of the eight degenerated bioprostheses that underwent redo surgery is fibrosis, calcification and thickening of the leaflets. After a change of our policy from 2017 to 2023, only two patients had prosthesis degeneration. Before proceeding to VIV-TAVR in Perceval, it is important to properly know its principal structural characteristics. This prosthesis is composed of a self-expandable Ni-Ti alloy stent and bovine pericardium. The stent is fully radiopaque and has the dual task of supporting the valve and fixing it in place; it is composed of an inflow ring and an outflow ring that are connected by columns and sinusoidal struts (Figure 6A–C). The bovine pericardium includes the tissue of the valve and the sealing collar, and it is composed of two sheets of pericardium that wrap the metal stent inside it axially to the supporting structures and around the inflow ring. The inflow ring is clearly visible under CT scan and fluoroscopy and constitutes our THV landing zone. The bovine pericardium that wraps the inflow ring will constitute the interface between the native annulus and the Perceval and between the Perceval and the THV. During the transfemoral procedure, one of the most tricky parts is to cross the Perceval both inside the outflow and the inflow ring. Especially in the case of ascending aorta enlargement, it can occur that the outflow ring is still detached from the aortic wall, and the guide wire can easily pass between it and the outflow ring and then inside the inflow ring. In this situation, it is not possible to advance the THV in place and it is mandatory to check for proper crossing (Figure 7). For this reason, we suggest to floss the full prosthesis with an inflated 12 or 14 × 20 mm balloon (Figure 4). If the inflated balloon is able to reach the left ventricle, the guide wire is in the correct position and it is possible to proceed with the next step of the THV implantation.

**Figure 8:**
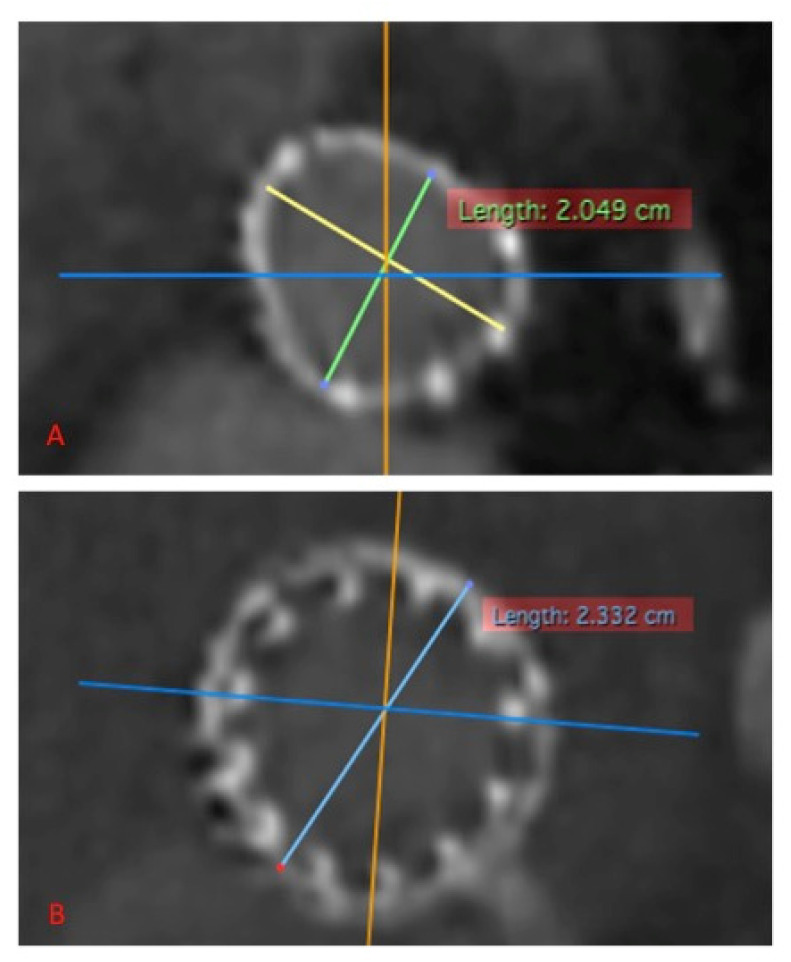
Preprocedural CT scan shows the inflow ring of a degenerated Perceval; a certain level of eccentricity is clearly visible with a sort of rounded triangular shape. (**A**) Post VIV-TAVIR CT scan of the same prosthesis, we can appreciate a circumferential expansion with minor diameter passing from about 2.05 cm to about 2.3 cm (**B**).

**Figure 9:**
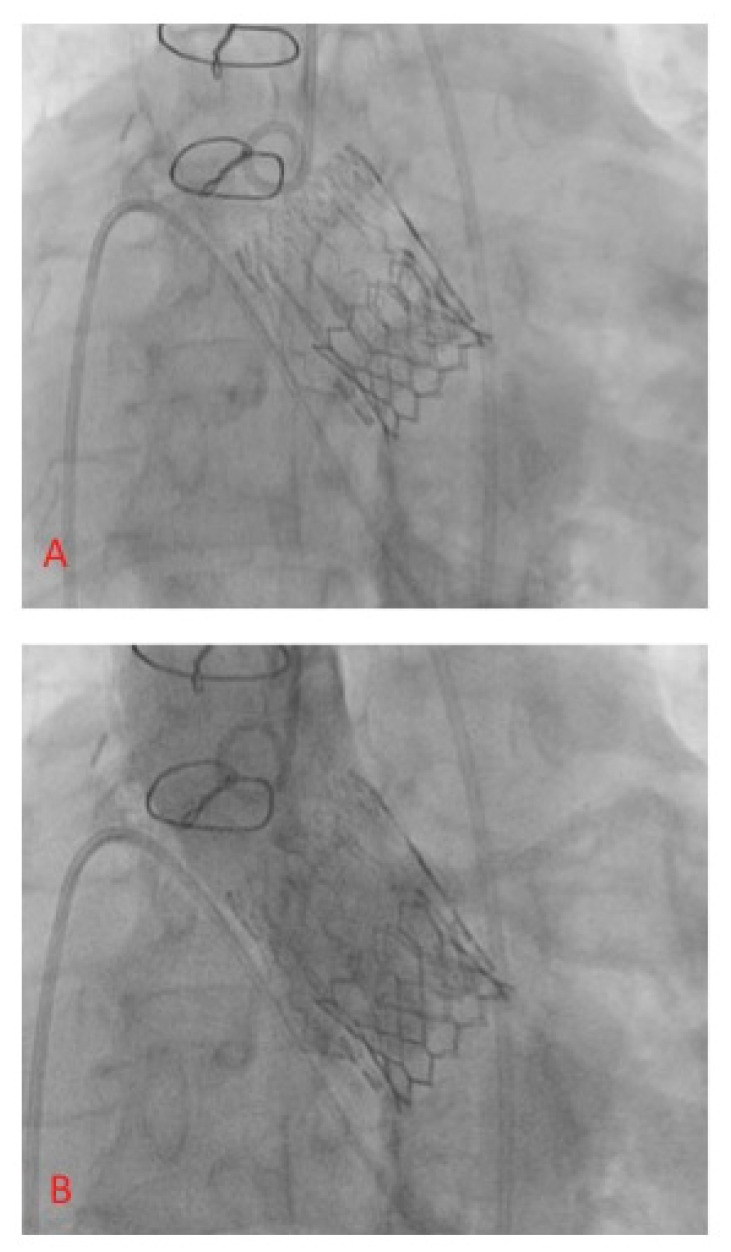
(**A**) THV delivery is not correct; going too deep in the ventricle may lead to suboptimal THV hemodynamic and earlier degeneration. (**B**) Angiography shows the absence of a paravalvular leak despite a deep delivery.

**Figure 10:**
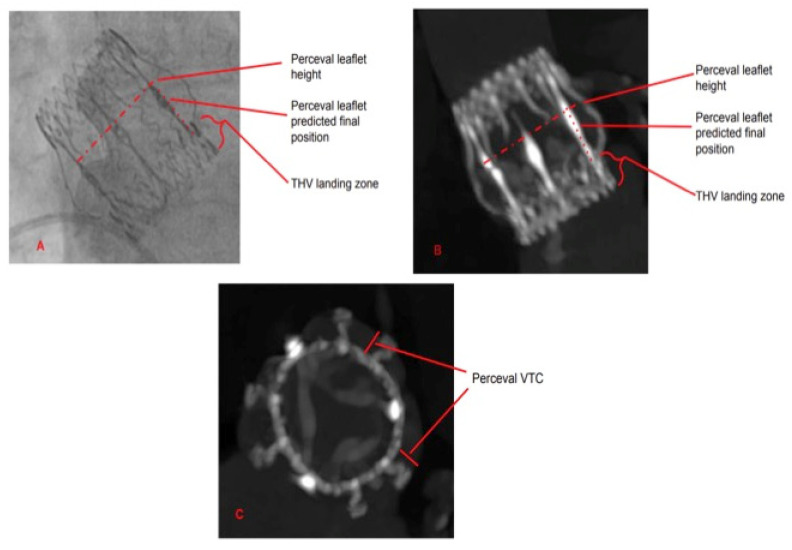
Radiological markers in Perceval. (**A**) Fluoroscopic view; (**B**) CT scan long axis view; (**C**) CT scan short axis view.

Valve-in-valve transcatheter aortic valve implantation for failed surgical bioprosthesis global experience is definitely increasing year after year [20,21]. In effect, it may offer a rapid and less invasive solution to bioprosthesis degeneration. There is a clear trend of increased use of VIV-TAVR compared to a relative reduction in the use of redo surgical replacement. Majmundar et al. confirm a good performance of VIV-TAVR with lower 30-day mortality in comparison to redo surgical replacement but a higher all-cause readmission rate and mortality in readmitted patients and similar secondary adverse events [21]. The two major adverse events that can compromise the outcome of VIV-TAVR and that are associated with an increased mortality rate and readmissions are substantial coronary artery obstruction and poor hemodynamic performance of the newly constrained THV. Careful selection of patients is mandatory to achieve good clinical performance and to avoid mortality and complications. To obtain this goal, preprocedural planning should forecast and consider how to manage any potential risk factors for coronary obstruction and for poor hemodynamic performance such as shallow and straight aortic root anatomy, low height of coronary artery take off, geometrical anatomy of the degenerated bioprosthesis, presence of globular calcifications, and small size of the surgical prosthesis [20]. Bioprosthesis valve fracture has been proposed to avoid post VIV-TAVR patients prosthesis mismatch that can occur in suboptimal expansion of a THV inside the surgical bioprosthesis [22,23,24]. In our series, we observed that Perceval, due to its self-expandable Ni-Ti alloy stent, keeps the capability of overexpansion up to 2.5 mm even after several years from the implantation and allows for circumferential expansion of the THV (Figure 8). This peculiar Perceval feature may help in preventing post VIV-TAVR patient prosthesis mismatch. To be honest, we have to highlight that, even in our series, we observed a higher postprocedural gradient in patients with a small prosthesis. However, no one is needed for extra post dilatation due to suboptimal hemodynamics. To gain the best THV hemodynamic performance, it is also important to have a proper THV delivery. It is fundamental to manage the height of implantation starting with the Sapien central marker at the level of the distal portion of the Perceval inflow ring (Figure 3) in order to achieve a correct alignment of the proximal edges of both prostheses once the THV is completely foreshortened (Figure 5), avoiding a delivery too deep in the ventricle (Figure 9A,B). However, in the case of deep delivery inside the ventricle, we did not observe any paravalvular leaks, maybe due to the relatively high landing zone offered by Perceval. The risk of coronary artery obstruction is easily predictable by measuring valve-to-coronary distance or virtual THV-coronary distance (VTC) and valve-to-sinotubular junction (VTSJ) distance at the preprocedural CT scan [25]. A VTC < 3 mm is considered to be at high-risk, 3 to 6 mm intermediate risk and > 6 mm low risk. Tarantini, Dvir and Tang have proposed an algorithm to manage VTC < 4 mm and narrow VTSJ in order to plan a conventional VIV-TAVR or to perform a BASILICA or to protect the coronary ostia [26]. In our series of cases, we never had to manage a coronary obstruction or plan a coronary ostia protection strategy. In effect, despite a relatively high leaflet height, Perceval can be considered a low risk prosthesis for this kind of complication due to several structural characteristics. VTC in Perceval is no longer a virtual distance but is a geometrical characteristic of the prosthesis that corresponds to the distance between the columns and the farthest point of the sinusoidal struts. Pericardial leaflets are located intra-annularly and mounted inside the columns system. The final position of the leaflet is predicted by an easily visible radiopaque marker (Figure 10A–C). The presence inside Perceval of several radiopaque markers that can clearly show THV landing zone height and dimensions, aortic root anatomy, final degenerated pericardial leaflet position and height could permit a completely no contrast medium procedure from CT scan planning to THV implantation. This could be extremely important for patients affected by severe renal failure or severe contrast medium allergies. Despite that, in case of very low coronary artery take off, shallow aortic root or very small or straight sinuses of Valsalva, even in Perceval, a VIV-TAVR can be a challenging procedure. A Perceval nitinol stent is composed of several critical structures that can interfere with the THV deployment and function. It is crucial to know the anatomy of the prosthesis in order to avoid any complications. In particular, the presence of the outflow ring must be considered during the phase of valve crossing for several reasons. It is not unfrequent to cross the valve outside the outflow ring (especially in the presence of ascending aorta dilatation), which is a typical pitfall of the valve-in-valve procedure in this type of prosthesis; it is mandatory to check for proper positioning of the guidewire inside the Perceval outflow ring before advancing the THV; in reverse, it is not possible to complete the THV deployment. Perceval is a high frame valve; when using a high frame self-expandable THV, it is crucial to obtain a perfect alignment of the two prostheses in order to avoid stent creep and THV dysfunction. In concomitance with a horizontal aorta, very high trans-prosthetic gradient and vessels tortuosity, it can be challenging to cross the degenerated leaflet and can require some more time and the use of special tools such as hydrophilic guidewire. Despite the fact that the VTC distance in Perceval is a geometric characteristic of the valve (that corresponds to the distance between the columns and the sinusoidal struts) and it is declared to be always > than 4 mm, it is always important to check the anatomy and the dimension of the aortic root and sinotubular junction. In fact, Perceval has a high pericardial leaflet height, and in the case of concomitant shallow and straight aortic root, it is not possible to exclude sinus sequestration and coronary ostia interference. For this reason, it is mandatory to properly analyze the preprocedural CT scan.

The mean transvalvular gradient at mid-term follow-up was 18.7 ± 5.3 mmHg. The rate of perioperative pacemaker implantation was 0%. Of the 32 patients, none had a preoperative permanent pacemaker. The durability of sutureless bioprosthesis at 10 years is comparable with sutured bioprosthesis, despite in the first phase of our experience (2011–2016) we often oversized the choice of the sutureless prosthesis. In a study by Johnston, actuarial estimates of explant of PERIMOUNT stented bioprostheses for structural degeneration at 10 and 20 years were 1.9% and 15% overall [27]. In our twelve years of experience, the rate of Perceval degeneration is 3.05%. We described the long-term follow-up of all Perceval implanted in our Center in a previous study [9].

Our experience with VIV-TAVR in degenerated Perceval sutureless showed favorable clinical and hemodynamic results at mid-term follow-up. Taking into account a series of precautions, complete planning with a CT scan, careful crossing of the prosthesis inside the outflow ring, and flossing the valve with a small balloon, Perceval can be considered an excellent prosthesis for a future valve-in-valve procedure for potential implant without contrast medium, presence of clearly visible radiopaque marker that facilitates THV alignment and positioning, capability of even late expansion and reduced the risk of interference with the coronary ostia.

## Figures and Tables

**Table 1:** Baseline patient characteristics.

Variables	Overall *n* = 32
Age, mean ± SD	79.6 ± 7.2
Sex, female, (%)	20 (62.5)
BSA, median (IQR), m^2^	1.6 (1.16–2.08)
Hypertension, (%)	26 (81)
Diabetes mellitus, (%)	9 (28)
COPD, (%)	11 (34)
Hyperlipidemia, (%)	21 (66)
Chronic renal failure, (%)	3 (9)
Peripheral vascular disease, (%)	4 (12.5)
EuroSCORE II, mean ± SD, (%)	9.5 ± 6.4
STS score, mean ± SD, (%)	8.7 ± 4.2

BSA, body surface area; COPD, chronic obstructive pulmonary disease; STS, Society of Thoracic Surgeons; SD, standard deviation.

**Table 2:** Intraprocedural data.

Variables	Overall *n* = 32	
Size degenerated Perceval		
	S (*n* = 5)	(16%)
	M (*n* = 11)	(34%)
	L (*n* = 14)	(44%)
	XL (*n* = 2)	(6%)
Isolated VIV-TAVR procedures		
	30	(94%)
Combined procedures	2	(6%)
- VIV-TAVR + PCI	1	(3)
- VIV-TAVR + VIV-TMVR	1	(3)
Surgical approaches		
Transfemoral	30	(94%)
Transapical	2	(6%)
Size TAVR		
20 mm	6	(19%)
23 mm	14	(44%)
26 mm	11	(34%)
29 mm	1	(3)

VIV, valve-in-valve; TAVR, transcatheter aortic valve replacement; PCI, percutaneous coronary intervention; TMVR, transcatheter mitral valve replacement.

**Table 3:** Postoperative data.

Variables	Overall *n* = 32
- At 30-day	
Mortality, *n*	0
Hospital stay, mean ± SD, days	4.6 ± 2.4
Vascular complications, *n* (%)	2 (6)
Permanent PMK, *n* (%)	0
- At follow-up	
NYHA, mean ± SD	1.25 ± 0.44
Permanent PMK, *n*	0
Stroke, *n*	1

ICU, intensive care unit; PMK, pacemaker; NYHA, New York heart association; SD, standard deviation.

**Table 4:** Echocardiographic findings of 32 degenerated Perceval.

Variables	Preoperative	At Discharge	At Follow-Up
LVEF, mean ± SD, (%)	56.2 ± 8.8	53.9 ± 4.3	53.2 ± 8
Mean pressure gradient, mean ± SD, (mmHg)	54.4 ± 15.4	16.8 ± 5.8	53.1 ± 18.3
Type of Perceval degeneration			- Stenosis, *n* (%) 6 (19)
			- Steno-regurgitation 22 (69)
			- Regurgitation 4 (12)

LVEF, left ventricular ejection fraction; SD, standard deviation.

**Table 5:** Echocardiographic findings of 32 VIV-TAVR.

Variables	At Discharge	At Follow-Up
LVEF, mean ± SD, (%)	52.8 ± 7.1	56.2 ± 5.4
Mean pressure gradient, mean ± SD, (mmHg)	19.1 ± 7.9	18.7 ± 5.3
Paravalvular leakage		
Trivial	1	1
Moderate	0	1

VIV, valve-in-valve; TAVR, transcatheter aortic valve replacement; LVEF, left ventricular ejection fraction; SD, standard deviation.

## Data Availability

Data available on request due to restrictions e.g., privacy or ethical. The data presented in this study are available on request from the corresponding author.
